# Welded Carbon Nanotube–Graphene Hybrids with Tunable Strain Sensing Behavior for Wide-Range Bio-Signal Monitoring

**DOI:** 10.3390/polym16020238

**Published:** 2024-01-15

**Authors:** Zixuan Hong, Zetao Zheng, Lingyan Kong, Lingyu Zhao, Shiyu Liu, Weiwei Li, Jidong Shi

**Affiliations:** 1 Center for Intense Laser Application Technology, College of Engineering Physics, Shenzhen Technology University, Shenzhen 518118, China2210412032@stumail.sztu.edu.cn (S.L.); 2 Frontiers Science Center for Flexible Electronics (FSCFE), Shaanxi Institute of Flexible Electronics (SIFE), Northwestern Polytechnical University (NPU), 127 West Youyi Road, Xi’an 710072, China; 3 Department of Materials Science and Engineering, Southern University of Science and Technology, Shenzhen 518055, China; 4 Chinese Laser Science (Shenzhen) Co., Ltd., Shenzhen 518106, China

**Keywords:** carbon nanotube, graphene, hybridization, strain sensor, bio-signal monitoring

## Abstract

Carbon nanotubes (CNTs) and graphene have commonly been applied as the sensitive layer of strain sensors. However, the buckling deformation of CNTs and the crack generation of graphene usually leads to an unsatisfactory strain sensing performance. In this work, we developed a universal strategy to prepare welded CNT–graphene hybrids with tunable compositions and a tunable bonding strength between components by the in situ reduction of CNT–graphene oxide (GO) hybrid by thermal annealing. The stiffness of the hybrid film could be tailored by both initial CNT/GO dosage and annealing temperature, through which its electromechanical behaviors could also be defined. The strain sensor based on the CNT–graphene hybrid could be applied to collect epidermal bio-signals by both capturing the faint skin deformation from wrist pulse and recording the large deformations from joint bending, which has great potential in health monitoring, motion sensing and human–machine interfacing.

## 1. Introduction

Flexible strain sensors have been studied extensively in recent decades, and hold great promise in bio-signal monitoring [1,2,3], human–machine interfacing [4,5], and soft robotics [6,7]. Usually, strain sensors are assembled by depositing a sensitive conductive film on a stretchable substrate, followed by mounting the electrodes at the ends of the film. The selection and processing of the conductive layer plays a dominant role in determining the performance of a strain sensor. Compared with metal films and nanowires, which have a high cost and strong tendency to corrode, carbon nanomaterials, including CNT and graphene, have been widely adopted as the sensitive layer of strain sensors [8]. The network of CNTs could respond to tensile strain via the interfacial sliding between nanotubes, which leads to the loss of overlapping joints and an overall increase in resistance [9,10,11]. Despite the moderate electrical response to strain over a wide range (>100%), the nanotube buckling upon strain release usually leads to a low sensitivity and linearity in cyclic tests. On the other hand, graphene with a continuous film structure could develop cracks upon tensile strain, which leads to sensitive electromechanical response but a low operational range and low durability in strain sensing [12,13,14].

To utilize the strength and circumvent the weakness of CNTs and graphene in strain sensing, the hybridization of the two components has been explored. The 1D CNTs and 2D graphene flakes in the nanocomposite could serve as complementary structural reinforcements, which leads to their having unique mechanical properties that differ from the individual components [15]. The CNT–graphene hybrids could be facially prepared by liquid-phase mixing [16,17], filtration [18,19], and sequential deposition [20,21], etc., and the hybrids have been widely applied for strain sensing [22,23] and selective molecular transport [20], as well as serving as the electrode material for electronic devices [24,25]. However, the low bonding strength between the CNT and graphene usually leads to insufficient coupling effects and a low stability at the contact interface, meaning that the mechanical strength and performance in functionality are usually below expectations. To enhance the bonding strength between CNT and graphene within the hybrid, Fang et al. developed a seamless CNT embroidered graphene hybrid film through using a CNT network as the template for graphene growth via chemical vapor deposition (CVD) [26,27]. The in situ hybridization of CNT and graphene forms a leaf-like composite film, and ensures a strong chemical bonding between the two components [28], through which the buckling of nanotubes upon cyclic stretch is prevented. However, the CVD growth of graphene is a self-limitation process [29,30]. When the copper substrate is fully covered by a graphene layer, the growth stops automatically for the isolation of feedstock (CH_4_) from catalytic substrate (copper). Therefore, the resulting graphene in the nanocomposite only has several atomic layers [31], which means that the reinforcement effect for the thick CNT layer is insufficient, and the dosage of CNT and graphene within the hybrid cannot be tailored. The preparation of a seamless CNT–graphene hybrid with an arbitrary ratio of components for strain sensing has yet to be developed.

In this manuscript, a novel methodology for preparing a CNT–graphene hybrid with an arbitrarily defined ratio of components was proposed and developed. Through the liquid-phase mixing of GO and CNT dispersions, followed by high-temperature annealing in H_2_ atmosphere, the GO could be reduced. Furthermore, the CNT and reduced GO could be welded together, resulting in a strongly bonded CNT–graphene hybrid. The as-prepared CNT–graphene hybrid could be transferred onto a stretchable polydimethylsiloxane (PDMS) substrate for strain-sensing, which demonstrates tunable electromechanical behavior by predefining the feedstock addition as well as the annealing temperature. The strain sensor based on the CNT–graphene hybrid could be applied as an epidermal sensor for recording various human signals and activities, with high potential for collecting primary data for Internet of Things (IoT) and artificial intelligence (AI) applications.

## 2. Materials and Methods

### 2.1. Preparation of the CNT–Graphene Hybrid

CNT ink (aqueous, 0.15 wt%, XFnano, 101944) and GO ink (aqueous, 0.2 wt%, XFnano, 100656, Nanjing, China) were mixed with vigorous stirring. Then, the mixed solution (1 g) was drop-casted on a copper foil (Alfa Aesar, Haverhill, MA, USA, Item No: 13382, 99.99%, 8 × 2 cm, 25 μm in thickness), before heating at 70 °C to evaporate the solvent. The CNT–GO ratio (in mass) was set at 45:1, 3:1, 1:3, 1:15 and 1:45 for different samples. Then, the Cu-supported CNT:GO hybrid film was loaded into a quartz tube for 30 min annealing under 20 sccm H_2_ flow and 60 sccm Ar flow. The annealing temperature was set at 500 °C and 1000 °C for different samples. Then, the quartz tube was naturally cooled to room temperature with the supply of the H_2_ and Ar flow.

### 2.2. Preparation of the CNT–Graphene-Hybrid-Based Strain Sensor

The base and curing agent of PDMS (Dow Corning Sylgard 184, Midland, MI, USA) were mixed in a 10:1 mass ratio to form the PDMS precursor. After degassing in vacuum oven, the precursor was poured onto the CNT/GO hybrid film covered Cu foil. Then, PDMS was cured at 70 °C for 2 h. The Cu/hybrid film/PDMS was soaked in FeCl_3_ aqueous solution (0.3 mol L^−1^) overnight to etch the Cu foil. The resulting hybrid film on PDMS was rinsed twice with distilled water to remove the FeCl_3_ residual and then dried in air. Silver paste (SPI FS05001) was added at the two ends of the hybrid film, and the end of the silver wires was submerged in the silver paste. After the evaporation of the solvent of silver paste, the silver wire was embedded in the solid-state silver paste for electrical measurement. Then, another layer of PDMS was coated on the silver paste for encapsulation, which ensures the stability of the electrode connection under tensile strain. The sensor is 4 × 1 cm in planar dimension, and the PDMS thickness is 1 mm.

### 2.3. Tests and Characterizations

Tensile stretch and real-time resistance recording in a strain-sensing test were conducted using a comprehensive flexible electronics testing platform (Sinoagg, AES-4SD, Beijing, China). The surface morphology of the hybrid was characterized by a scanning electron microscope (SEM, Carl Zeiss, GeminiSEM 300, Oberkochen, Germany). The stress–strain curve of the hybrid was obtained by a nano-indenter (KLA, G200, Milpitas, CA, USA). The contact angle test was conducted using a contact angle meter (Dingsheng, SDC-350, Yangzhong, China). Raman spectrum was obtained by a Raman spectroscopy (Renishaw inVia, Wotton, UK). X-ray Diffraction (XRD) pattern was obtained by an X-ray diffractometer (Rigaku, Smartlab, Tokyo, Japan). The thickness of the film was measured by removing the Cu substrate with an FeCl_3_ etchant, and using a silicon wafer to scoop up the film. After rinsing the film with deionized water and drying, the height profile at the film–substrate interface was acquired using a laser confocal microscope (Sensofar, S neox090, Barcelona, Spain), and the film thickness could be obtained using the step height at the film–substrate interface.

## 3. Results and Discussion

### 3.1. Preparation of CNT–Graphene Hybrid

**Figure 1:**
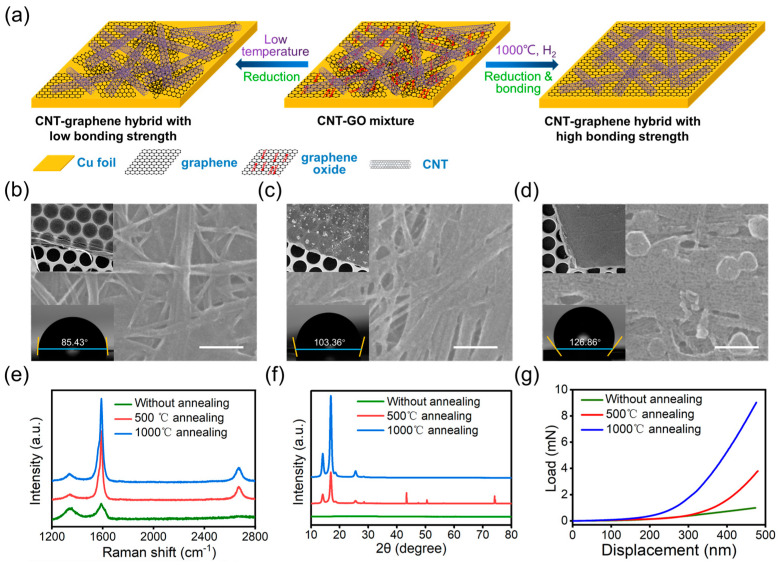
Preparation and characterization of the CNT–graphene hybrid. (**a**) The schematic illustration of the preparation process. (**b**–**d**) The morphology of the hybrid before (**b**), after 500 °C (**c**) and after 1000 °C annealing (**d**). The corresponding low-magnification images and contact angle tests are on the top left inset and bottom left inset of each figure. (**e**) The Raman spectroscopy of the hybrid before, after 500 °C and after 1000 °C annealing. (**f**) The XRD patterns of the hybrid before, after 500 °C and after 1000 °C annealing. (**g**) The stress–strain curve of the hybrid before, after 500 °C and after 1000 °C annealing. For the morphological and structural characterization in (**b**–**g**), the initial CNT:GO = 3:1.

Figure 1a demonstrates the preparation process of the CNT–graphene hybrid. The mixture of CNT and GO dispersion was drop-casted on copper (Cu) foil, followed by annealing in H_2_ atmosphere. As the common substrate for the CVD growth of graphene [30,32], Cu was selected as the substrate, with its hexagonal crystalline lattice, which is similar to the structure of graphene. Furthermore, the lattice parameter of the (111) plane for Cu is 2.56 Å, which is close to that of graphene (2.46 Å), which favors the epitaxial nucleation and growth of graphene on the Cu surface [33,34]. The low solubility of carbon atoms in Cu also facilitates the control of layer numbers of graphene [30,35]. The high-temperature annealing of the CNT–GO hybrid could not only reduce GO to rGO in situ, but also activate the carbon source to achieve better crystallinity as well as a strong bonding between CNT and graphene in the hybrid [28]. The H_2_ atmosphere not only plays a major role in the reduction of GO, but also protects the Cu substrate from corrosion at high temperatures, which is indispensable in the annealing process. Within the pristine CNT–GO hybrid, the voids of CNT network were partially covered by GO flakes (Figure 1b). The majority of pores within the network were maintained, through which the hybrid film is transparent under the electron beam (Figure 1b, top inset). After annealing, the porosity of the hybrid decreased dramatically, with the nanotubes tightly bundled together with each other (Figure 1c,d). However, the compactness of the hybrid film increases dramatically with the annealing temperature, resulting in high opaqueness for the electron beam (Figure 1c,d, top inset). The loss of oxygen-containing groups during the annealing process leads to an increase in the hydrophobicity of the hybrid film, As a result, the contact angle of the surface increases from 85.43° in unannealed state to 103.36° with 500 °C annealing and 126.86 for 1000 °C annealing (Figure 1b,d, bottom inset). The thickness of the pristine CNT–GO film is ~1.028 μm, which slightly decreases with 500 °C annealing, and increases a little with 1000 °C annealing (Figure S1). We attributed the change in film thickness to the combined effect of the loss of oxygen-containing functional groups (occuring at a low annealing temperature) and the nucleation and growth of graphene (occuring at a high annealing temperature). Furtherore, we inferred that the film thickness of the film is not changed during the etching of Cu, as the CNT and graphene does not react with FeCl_3_ etchant.

### 3.2. Structural Characterization of CNT–Graphene Hybrid

To investigate the structural characteristics of the CNT–graphene hybrid, Raman spectroscopic and XRD characterizations were conducted. For the pristine hybrid of CNT:GO = 1:3 in mass, the Raman spectrum shows typical GO bands at around 1340 cm^−1^ and 1590 cm^−1^ (Figure 1d) [36,37]. However, the 2D band for sp2 carbon nanomaterials (graphene and CNT) does not appear, indicating that the spectroscopic traits of CNTs are screened by GO. Analogously, the XRD spectrum of the pristine hybrid shows no distinctive spikes, but only a wide bulge within 2θ = 20–30° (Figure 1e and Figure S2), which suggests that GO is amorphous in the hybrid. After annealing, the crystallographic characteristics of sp2 carbon appear in both the Raman and XRD spectra (Figure 1d,e), indicating the elimination of oxygen containing groups in GO as well as the formation of highly ordered crystalline graphene [38,39,40]. Mechanical tests of the hybrid before annealing, after 500 °C annealing and after 1000 °C annealing were also conducted. The modulus of the hybrid increases with annealing temperature, which could be attributed to the increased bonding strength between CNTs after the chemical hybridization by graphene. Furthermore, the strong bonding dramatically increases the reinforcement of graphene to the CNT network, which creates a compact structure with a seamless CNT–graphene linkage. The morphological, structural and mechanical characterizations indicate that a strongly bonded CNT–graphene hybrid could be prepared through the high-temperature annealing of a CNT–GO hybrid. The welding process between CNT and graphene could be attributed to the unzipping of CNTs at a high temperature [28,41], and the exposed hexagonal carbon lattice matches the crystalline structure of graphene film. Since the CNT could be etched by H_2_ during the annealing process [42], and the generated active carbon atoms could nucleate on Cu foil and serve as the “stitching agent” for the exposed CNT surface and graphene surface, which induces the welding and chemical bonding at the CNT–graphene interface. Compared with the CVD growth of graphene on CNT-coated Cu foil, the resulting CNT–graphene hybrid obtained using this strategy has tunable properties; the ratio of CNT and GO dosage can easily be varied in the deposition process, as well as via the setting of a specific annealing temperature, which could potentially fulfill the requirements in various application scenarios.

### 3.3. Strong Bonding between CNT and Graphene in the Hybrid Obtained via Annealing

**Figure 2:**
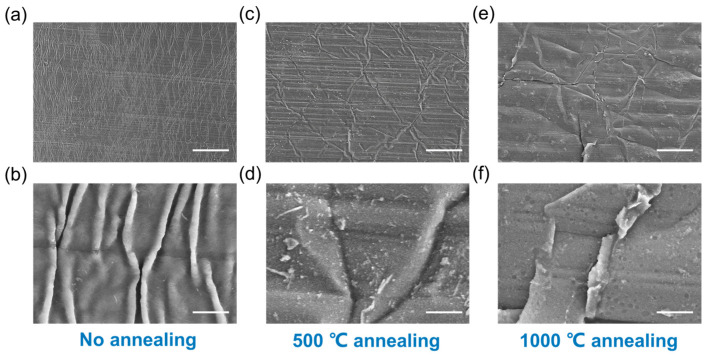
The morphology of CNT–graphene hybrid (initial CNT:GO = 45:1) after a cyclic stretch of 10%. (**a**,**b**) The low- (**a**) and high-magnification (**b**) SEM images of the hybrid without annealing. (**c**,**d**) The low- (**a**) and high-magnification (**b**) SEM images of the hybrid after 500 °C annealing. (**e**,**f**) The low- (**a**) and high-magnification (**b**) SEM images of the hybrid after 1000 °C annealing. Scale bar for (**a**,**c**,**e**): 50 μm. Scale bar for (**b**,**d**,**f**): 2 μm.

As discussed in previous reports, the CNT network develops wave-like buckles upon cyclic stretching due to its intrinsic flexibility and weak bonding at the nanotube joints [10,26]. The reinforcement of the CNT network by in situ graphene hybridization could enhance the rigidity of the network and restrain the formation of buckles. However, the reinforcement relies on a strong chemical bonding between CNT and graphene, which cannot be achieved by the mechanical mixing of the two components. To investigate the effect of annealing on the bonding strength of CNT and graphene, the morphologies of the as-prepared hybrid before and after annealing under cyclic stretch were compared. For the hybrid of CNT:GO = 45:1 in mass, there is a high density of surface wrinkles perpendicular to the stretching direction after a cyclic tensile strain of 10% (Figure 2a,b), which indicates that the stiffness of the hybrid is mainly dominated by CNTs due to the low graphene ratio and poor CNT–graphene coupling. For the hybrid annealed at 500 °C after cyclic stretching, the density and height of the wrinkles shows a dramatic decrease (Figure 2c,d), and cracks also generate amongst the film (Figure S3), which indicates a higher film stiffness and stronger interaction between CNT and graphene. When the annealing temperature reaches 1000 °C, there are very few folds, with a low amplitude and in random orientation (Figure 2e,f), and the cracks are obviously wider, along with the local delamination from the substrate (Figure 2f), through which the reinforcement effect further improves at such a high temperature. The above result verifies that the CNT network could be effectively reinforced by hybridized graphene after annealing, and a strongly bonded CNT–graphene hybrid could be achieved using this strategy.

### 3.4. Strain-Sensing Performance of the CNT–Graphene Hybrid

**Figure 3:**
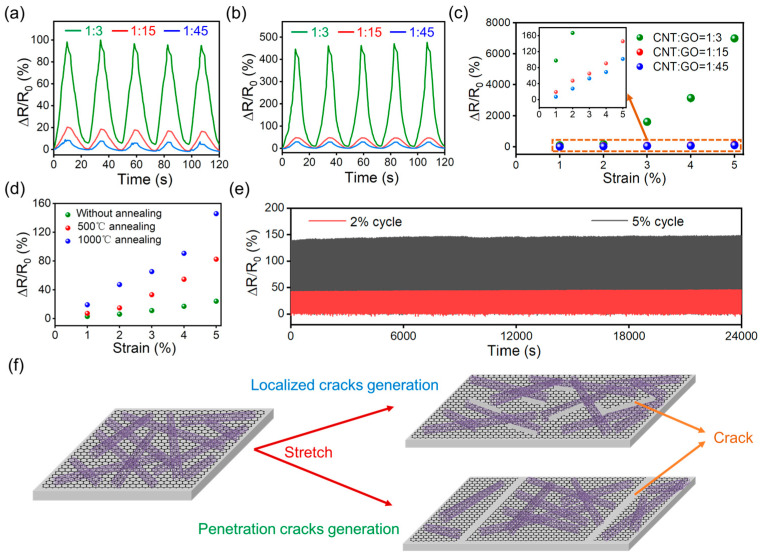
The strain-sensing performance of the CNT–graphene hybrid. (**a**) The resistance response of the hybrid after 1000 °C annealing with different initial CNT/GO ratios upon a cyclic strain of 1%. (**b**) The resistance response of the hybrid after 1000 °C annealing with different initial CNT/GO ratios upon a cyclic strain of 2%. (**c**) A comparison of the sensitivity of the hybrids with different CNT/GO ratios. (**d**) A comparison of the sensitivity of the hybrid with different annealing processes (initial CNT:GO = 1:15). (**e**) The electrical response of the sensor (initial CNT:GO = 1:15, 1000 °C annealing) 1000 cyclic stretches, with a tensile strain of 2% and 5%. (**f**) The schematic illustration of the crack-generation mechanism for CNT–graphene hybrid with weak (top) and strong (bottom) bonding.

The strain-sensing performance of the CNT–graphene hybrid is investigated based on the CNT–graphene ratio, as well as the effect of annealing. For the hybrid annealed at 1000 °C with initial CNT:GO = 1:3, 1:15 and 1:45, a repeatable and monotonic piezoresistive response to the applied strain is achieved, without abnormal spikes within the small strain range for CNT-based strain sensors, and the sensitivity decreases with the graphene ratio (Figure 3a,b). The gauge factor (GF) value is widely adopted to evaluate the sensitivity of a strain sensor. For the sensors in this work, the electromechanical response is not linear, indicating a GF that varies with strain. Therefore, we used the average GF, which is calculated by dividing the relative resistance change (ΔR/R_0_) by the applied strain (ε), to describe the sensitivity of the strain sensors [43,44]. The average GF of the CNT:GO = 1:3, 1:15 and 1:45 hybrid for 5% strain is 1399, 29.16 and 20.34, respectively (Figure 3c), which is comparable to the state-of-the-art carbon-nanomaterials-based strain sensors [45,46,47]. On the other hand, the strain-sensing performance of the hybrids with CNT:GO = 1:15 before annealing, after 500 °C annealing, and after 1000 °C annealing were also compared (Figure 3d), with the electrical response to strain increasing with annealing temperature. Also, the durability of the sensor was investigated by the repeated loading of 2% and 5% tensile strains 1000 times. The electrical response is rather stable during the cycles, indicating the high cyclic stability of the sensor (Figure 3e). The effect of annealing on the strain-sensing properties could be attributed to the tendency of crack generation and propagation due to the increased film stiffness (Figure 3f). Before annealing, CNT and GO loosely overlap, with a strong tendency toward interfacial sliding upon stretch. The cracks are usually generated locally at the CNT/GO interface, which is short and narrow (Figure S4a), leading to a small increase in resistance. The stiff CNT–graphene hybrid usually develops sharp penetration cracks upon stretching after annealing, validated by the pullout of CNTs at the fracture surface (Figure S4b) [48]. The crack generation process upon stretch is the underlying reason for the non-linear electromechanical response [49,50]. Additionally, the reliance of average GF on the initial CNT–GO ratio, as well as the annealing temperature, applies to all samples in our work (Table S1). Therefore, through tuning the initial CNT/GO content and setting the annealing temperature, the strain-sensing properties of the hybrid film could be facilely tailored, which could fulfill the requirements for different application scenarios.

### 3.5. The Application of CNT–Graphene-Hybrid-Based Strain Sensor for Bio-Signal Monitoring

**Figure 4:**
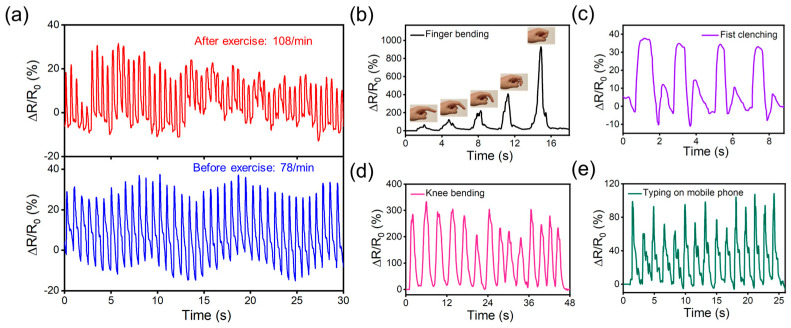
The application of CNT–graphene hybrid-based strain sensors for monitoring physiological signals and biological activities. (**a**) The real-time resistance recording of the sensor in response to pulse vibration before (bottom) and after (top) exercise. (**b**) The response of the sensor to finger-bending at different angles. (**c**) The response of the sensor to fist-clenching. (**d**) The response of the sensor to knee-bending. (**e**) The resistance recording of tapping the screen of mobile phones.

Owing to the excellent strain sensing performance of the CNT–graphene hybrid, it can be applied to capture various bio-signals of human beings when mounted on skin. Since the skin and CNT–graphene hybrid film were separated by the insulated PDMS substrate, the sensor’s signal could only be induced from the strain through the deformation of skin, without the influence of bioimpedance and bioelectricity. The CNT–graphene-hybrid-based strain sensor was attached to the radial artery of a 25-year-old male adult to record the wrist pulse. The sample with CNT:GO = 1:3 was used, as it had the highest sensitivity and could catch the faint pulse signal. When the tester is in a resting state, a highly repeatable pulse waveform was recorded during the 30 s testing, with the heart rate determined to be 78 min^−1^. Notably, both the percussion (P-) and tidal (T-) wave could be clearly identified, validating the high resolution of the strain sensor and its ability to catch small deformations (Figure 4a). On the other hand, after the tester did some exercise, the heart rate dramatically increased to 108 min^−1^ from the real-time resistance recording. Furthermore, the pulse amplitude dramatically fluctuates with a blurred T-wave, which is similar to previous reports [3]. The recording of an arterial pulse at a high resolution shows that our sensor has high potential in wearable healthcare. In addition, the sensor could also be tied to the finger pulp to catch the fingertip pulse, which induces much less skin deformation compared to the wrist pulse [51,52] (Figure S5). The heart rate caluclated from the real-time resistance recording agrees with that measured by the smart watch. For the monitoring of muscle and joint motions, which induces dramatically higher deformation, the sample with CNT:GO = 1:15 was adopted due to its adequate sensitivity and low tendency to generate cracks. When the sensor was attached to the proximal interphalangeal (PIP) joint of the index finger, the piezoresistive response monotonically increases with the bending angle of the finger (Figure 4b). Furthermore, the sensor could be adhered onto the back of the hand (Figure S6a). When the tester clenched his fist, the skin on the hand back was stretched, and a characteristic waveform was generated. This waveform is highly repeatable during multiple clenching–relaxation cycles (Figure 4c). Furthermore, the sensor could also reliably catch the large deformation at the knee joint (Figure S6b), with the film resistance increasing several times upon knee-bending, and returning to the original state when the knee is straightened (Figure 4d). The sensor could also record the tapping of a mobile phone by the thumb when adhered to the first dorsal interossei muscle on hand (Figure S6c). Each tapping process generated a spike, with the amplitude and waveform varying based on the tapping force and modes (Figure 4e). Based on the results presented above, the CNT–graphene hybrid develped by our strategy is capable of recording full-range human activities at a high resolution, which makes it promising in biomedical monitoring and human–machine-interaction applications.

## 4. Conclusions

In conclusion, a novel strategy to achieve a strongly bonded CNT–graphene hybrid has been raised by liquid-mixing CNT and GO dispersions, followed by the in situ reduction of GO through thermal annealing. The reinforcement of graphene to the CNT network was validated by the increased overall film modulus, as well as the prevention of film wrinkling upon cyclic stretching. The bonding strength between CNT and graphene, as well as the resulting film stiffness, could be tailored by predefining the ratio of the components and the annealing temperature, which leads to specific strain-sensing performances, and could be further applied in various bio-signal monitoring scenarios. The CNT–graphene hybrid-based strain sensor could not only capture small deformations from arterial pulses at the wrist and fingertip, but also record large deformations from the bending of various joints in the human body, which offers it great potential in the collecting of primary biomedical data for daily health monitoring and motion sensing. The ease of preparation, tunable strain-sensing properties, as well as the reliable recording of various human activities, offers our strategy great promise in the building of smart electronics.

## Data Availability

Data are contained within the article and Supplementary Materials.

## Supplementary Materials

The following supporting information can be downloaded at: https://www.mdpi.com/article/10.3390/polym16020238/s1, Figure S1: The height profile for the film before annealing, after 500 °C annealing and after 1000 °C annealing, transferred onto a silicon wafer at the film–substrate interface; Figure S2: The XRD pattern of the CNT–GO hybrid before annealing; Figure S3: The magnified view of the hybrid after 500 °C annealing to reveal the distribution of cracks; Figure S4: The morphology of the fracture surface for the hybrid with and without annealing; Figure S5: The recording of fingertip pulse by the sensor; Figure S6: The photos showing the sensor adhered to the back of the band, at the knee joint, and on the first dorsal interossei muscle of the hand. Table S1: The gauge factor value of the CNT/graphene film with varying initial CNT:GO ratios and different annealing processes.
