# Supplementary material for: Welded Carbon Nanotube–Graphene Hybrids with Tunable Strain Sensing Behavior for Wide-Range Bio-Signal Monitoring
Source: Polymers (Basel). 2024 Jan 15;16(2):238. doi: 10.3390/polym16020238 (PMC10819715; doi:10.3390/polym16020238)
Supplement: Supplementary file 1 [file polymers-16-00238-s001.zip › polymers-2775865-supplementary.pdf]

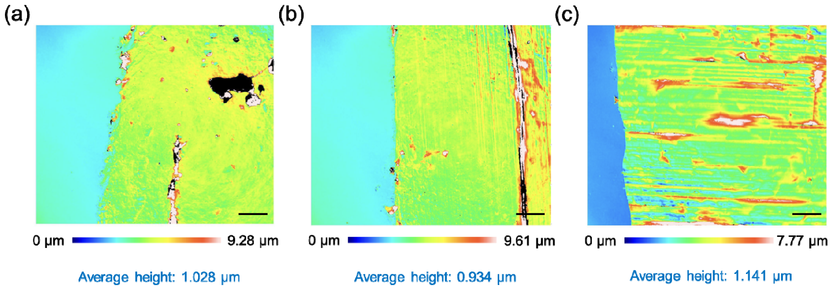

Figure S1. The height profile for the film before annealing (a), after 500 °C annealing (b) and after 1000 °C annealing (c) transferred on silicon wafer at the film-substrate interface. The average height of the film is automatically calculated by the software.

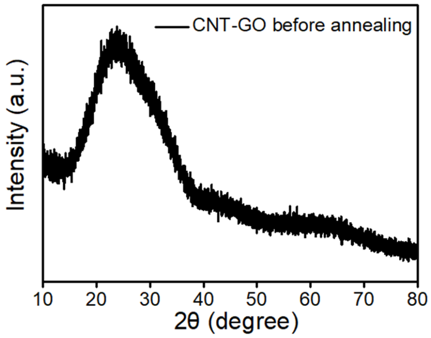

Figure S2. The XRD patterned of CNT-GO hybrid before annealing (magnified view of Figure 1e).

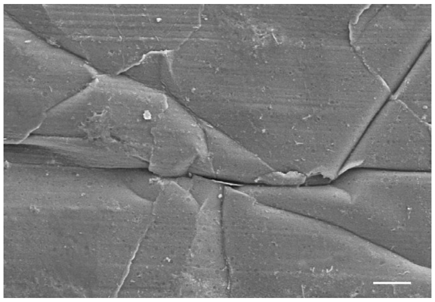

Figure S3. The magnified view of the hybrid after 500 °C annealing to reveal the distribution of cracks. Scale bar: 5μm.

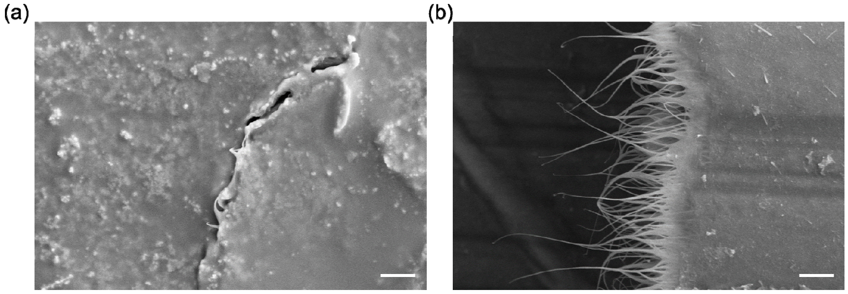

Figure S4. The morphology of the fracture surface for the hybrid without (a) and with annealing. Scale bar for (a): 2 μm. Scale bar for (b): 1 μm.

	No annealing	500 °C annealing	1000 °C annealing
CNT:GO=45:1	82.1	180.4	326.1
CNT:GO=3:1	22.81	48.16	176.8
CNT:GO=1:3	13.12	29.04	97.98
CNT:GO=1:15	2.92	7.204	19.12
CNT:GO=1:45	1.67	3.788	6.635

Table S1. The gauge factor value of the CNT/graphene film with varied initial CNT:GO ratio and different annealing process.

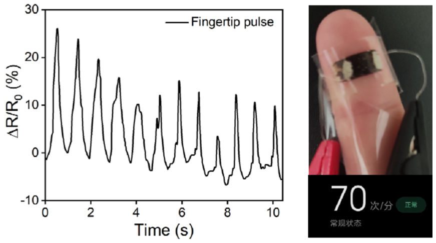

Figure S5. The recording of fingertip pulse by the sensor (left), with the heart rate agreeing with that measured by a smart watch (right).

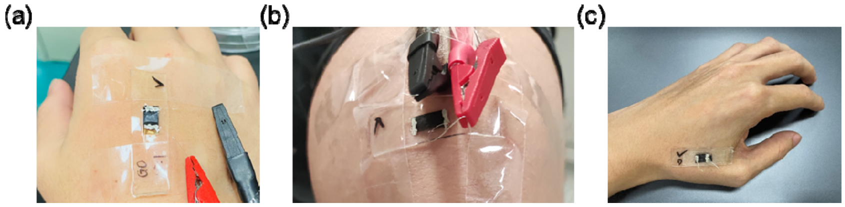

Figure S6. The photos showing the sensor adhered on the back of the band (a), at the knee joint (b) and on the first dorsal interossei muscle on hand (c).
